# Pregnancy and postpartum experiences of women undergoing hemodialysis: a qualitative study

**DOI:** 10.1590/2175-8239-JBN-2022-0001en

**Published:** 2022-09-30

**Authors:** Débora Bicudo Faria-Schützer, Anderson Borovac-Pinheiro, Larissa Rodrigues, Fernanda Garanhani Surita

**Affiliations:** 1 Universidade Estadual de Campinas, Faculdade de Ciências Médicas, Departamento de Tocoginecologia, Campinas, SP, Brazil.

**Keywords:** Renal Dialysis, Renal Insufficiency, Chronic, Postpartum Period, Pregnancy, Qualitative Research, Diálise Renal, Insuficiência Renal Crônica, Período Pós-Parto, Gravidez, Pesquisa Qualitativa

## Abstract

**Introduction:**

There are particularities of chronic kidney disease (CKD) in women and their treatment. The biology of women exposes them to greater risk factors for CKD and both pregnancy and the postpartum period place an additional burden on renal health. Pregnancy complications may cause or worsen CKD.

**Objective:**

To explore the experiences of women with CKD undergoing hemodialysis in relation to their reproductive history.

**Methods:**

This study consisted of clinical-qualitative design with semi-structured individual interviews and open-ended questions. The sample selection was intentional and according to the theoretical saturation criterion. The data analysis was carried out based on the seven steps of the clinical-qualitative content analysis and validated by Nvivo11. This study was conducted in a public hemodialysis clinic of the Brazilian National Health System.

**Results:**

Twelve women undergoing hemodialysis were interviewed. The results from the analysis revealed three categories: 1) Association of pregnancy with CKD; 2) Nebulosity in relation to diagnosis and reproductive history 3) Being a woman undergoing hemodialysis.

**Conclusion:**

Our study showed the importance of considering the specificities of CKD in women, suggesting that these issues are important for diagnosis and treatment adherence. Consideration of reproductive life history allows the health of women undergoing hemodialysis to be promoted holistically, including aspects of mental health.

## Resumo

**Introdução:**

Existem particularidades da doença renal crônica (DRC) em mulheres e seu tratamento. A biologia das mulheres as expõe a fatores de risco mais elevados para DRC e tanto a gravidez quanto o puerpério implicam um ônus adicional à saúde renal. Complicações na gestação podem causar ou piorar a DRC.

**Objetivo:**

Explorar as experiências de mulheres com DRC submetidas à hemodiálise em relação ao seu histórico reprodutivo.

**Métodos:**

Este estudo consistiu em desenho clínico-qualitativo com entrevistas individuais semiestruturadas e questões abertas. A seleção da amostra foi intencional e de acordo com o critério de saturação teórica. A análise de dados foi realizada com base nos sete passos da análise clínico-qualitativa de conteúdo e validada pelo Nvivo11. Este estudo foi realizado em uma clínica pública de hemodiálise do Sistema Único de Saúde brasileiro.

**Resultados:**

Foram entrevistadas 12 mulheres em hemodiálise. Os resultados da análise revelaram três categorias: 1) Associação da gravidez com DRC; 2) Nebulosidade em relação ao diagnóstico e à história reprodutiva; 3) Ser mulher e fazer hemodiálise.

**Conclusões:**

Nosso estudo mostrou a importância de considerar as especificidades da DRC em mulheres, sugerindo que estas questões são importantes para o diagnóstico e a adesão ao tratamento. A consideração do histórico de vida reprodutiva permite promover de forma holística a saúde das mulheres submetidas à hemodiálise, incluindo aspectos de saúde mental.

## Introduction

In this study, we approach women's experiences in relation to hemodialysis and their reproductive life. There are specificities in women's health that must be considered to improve their quality of life: especially socio-emotional care, which can be provided by health teams. In addition, listening to the history of the reproductive lives of these women allows for a greater understanding of the importance of pregnancy and the postpartum period as a window of opportunity for diagnosing diseases, including kidney disease, which can manifest or worsen^1^.

Approximately 1.2 million people in the world die because of kidney disease^2^. Chronic kidney disease (CKD) is a prolonged and insidious disease that is asymptomatic for most of its evolution^3,4,5^. The incidence of CKD has increased year by year, affecting approximately 3% of women of fertile age^2^. Besides the risk factors that are also common in the male population, such as diabetes mellitus and hypertension, women are exposed to greater risk factors, like urinary tract infections. In addition, pregnancy places an additional burden on the health of women and there is the possibility that complications during pregnancy could cause kidney injuries^6^.

An important treatment for terminal CKD is hemodialysis. This treatment also has a considerable impact on the patients’ quality of life, as it requires frequent procedures, with three or more clinical appointments per week. As in similar situations in chronic diseases, social contacts are established with groups of people who share the same illness and new connections are made^7,8,9^. In advanced stages, CKD causes a reduction in fertility, menstrual disorders, and amenorrhea^10^. Unfavorable maternal and perinatal outcomes are frequent among women with CKD^11,12^.

For women with CKD, the decision to become pregnant can be emotionally complicated due to the inherent health risks, the strain on the family, and the perceived risk of fetal loss. Multi-disciplinary care, including nephrologists, gynecologists, and psychological support, is recommended^13^.

On the other hand, there is a group of women who suffer from physiological overload due to pregnancy, which gives rise to a process of progressive loss of renal function that develops into terminal CKD^1,14^. Other pregnant women with complications such as preeclampsia, placental abruption, or other hemorrhages may present changes in renal function that may also evolve into CKD^1^. Pregnancy is a cardiometabolic and renal stress test for women with or without CKD and clearly a high-risk situation for women with pre-terminal or terminal CKD^15,16^.

With this in mind, the objective of this research was to explore the experiences of these women undergoing hemodialysis in relation to their reproductive histories in order to identify issues in the interviewees’ perception and to raise possible themes to be explored in the literature on women's health and nephrology.

## Material and Methods

### Study Design

We used a clinical-qualitative method based on the concepts of qualitative research in healthcare settings, with the following particularities: the existential attitude in valuing elements of anguish and anxiety in the existentiality of the subject under study; the clinical attitude of the participant seeking protection from her emotional suffering; and lastly, the psychoanalytic attitude, which results from the conceptions emerging from the dynamics of the participant's unconscious^17,18^.

### Research Setting

This study was conducted in a public hemodialysis healthcare clinic of the Brazilian National Health System in southeastern Brazil. This healthcare service has a multi-professional and interdisciplinary team with nurses, physicians, psychologists, nutritionists, and social workers. The clinic serves 400 patients, 40% of whom are women.

### Sampling

The selection of participants was intentional, and the number of participants was defined by the criterion of theoretical saturation, when it becomes clear that “new elements to subsidize the desired (or possible, under the circumstances) theorization are no longer surmised in the field of observation”.

The criteria for inclusion in the sample were: women over 18 years of age with a relationship between reproductive history and terminal CKD, who were undergoing hemodialysis, and who had been identified in a preliminary observational study at the same site.

### Data Collection

A period of acculturation in 2019 preceded data collection between September 2019 and March 2020.

The data was collected by the first author (DBFS), a psychologist, in a suitable setting at the same healthcare facility frequented by the interviewees, thus allowing the interviews to take place in the same clinical setting. Clinical-qualitative criteria were followed: establishing rapport, explanations about the theme and aims of the research, collection of identification data, and the request for permission to use a recorder. Observations about the behavior of the interviewee were registered in the field journal.

To make the clinical-qualitative method viable, we used semi-structured in-depth interviews, with open-ended questions, developed from a basic but not rigid script. This allowed the interviewer to make the necessary adaptations based on the information given by the interviewees, who were allowed to speak freely about their experiences. The interview that began in this way was planned based on a script with questions that were determined by the aim of the study and that provided support for the researcher.

- How was your pregnancy?
- Do you notice any association between pregnancy and CKD?
- Tell me a little about your life and your gestational history.
- What feelings did you have when you received some news of kidney disease in pregnancy?
- How was your life after pregnancy in relation to CKD?
- How is it for you to live with a CKD?
- Does CKD interfere with your daily life? How?

The interviews were later transcribed in their entirety and formed was is known as the corpus. This was carried out by the researchers and an assistant trained by the researchers.

### Data Analysis

The data analysis was carried out based on the seven steps of the clinical-qualitative content analysis^18^: 1) Editing of material for analysis (DBFS); 2) Suspended attention (DBFS and LR); 3) Construction of units for analysis (DBFS, LR and FGS); 4) Construction of sense codes (DBFS, LR and FGS); 5) Construction of categories (all authors); 6) Validation (all authors); 7) Discussion (all authors).

NVivo 11 (QSR International, Melbourne, Australia) was used for validation. The COREQ checklist^19^ was used to promote good qualitative rigor in the study.

### Ethical Approval

This study complied with the National Health Council Resolution on health research with human beings and was approved by the Ethics Committee of the School of Medical Sciences of the State University of Campinas. All participants signed the informed consent before the interviews.

## Results

Twelve women were interviewed. Some of their socio-demographic and health charactaristics are described in Table 1.

**Table 1.** Biodemographic characteristics of the participants, 2021

Interviewees	Age	Fixed companion	Cause of disease	Years in hemodialysis	Number of births	Number of miscarriages	Years between last pregnancy and CKD diagnosis
**I1**	63	Yes	AH + UTI	24	4	1	10
**I2**	23	Yes	UTI + Lithiasis	<1	1	0	3
**I3**	49	Yes	unknown	26	1	0	3
**I4**	32	Yes	UTI + Nephritis	19	1	0	CKD prior to last pregnancy
**I5**	41	Yes	Drug addiction	<1	2	0	CKD started in last pregnancy
**I6**	32	Yes	AH	7	1	0	1
**I7**	60	No	AH	3	5	0	20
**I8**	38	No	AH + Nephritis	2	2	0	6 (with 1 pregnancy after diagnosis)
**I9**	34	No	AH + DM	4	1	1	4 (with 1 pregnancy, 1 miscarriage after diagnosis)
**I10**	47	No	AH	9	2	2	15
**I11**	40	Yes	AH + UTI + Reflux	20	1	2	CKD prior to all pregnancies
**I12**	69	No	AH	17	9	0	15

CKD = Chronic Kidney Disease; AH = Arterial Hypertension; UTI = Urinary Tract Infection; DM = Diabetes Mellitus.

**Figure 1.**
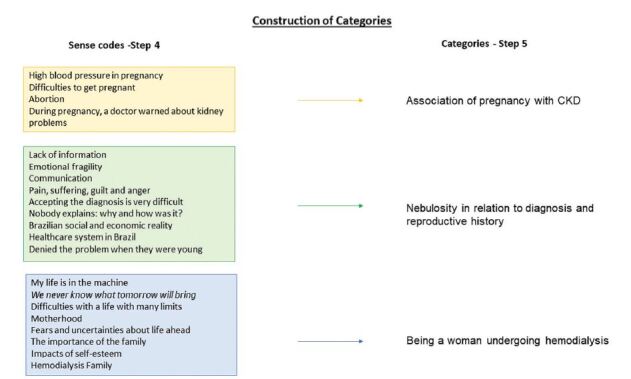
Process of data analysis and its relation with the research questions.

Through the analysis of the clinical-qualitative content, we identify three main categories: 1) Association of pregnancy with CKD; 2) Nebulosity in relation to diagnosis and reproductive history 3) Being a woman undergoing hemodialysis. The process of constructing categories through the analysis of qualitative content is shown in Figure 1.

**Figure 2.**
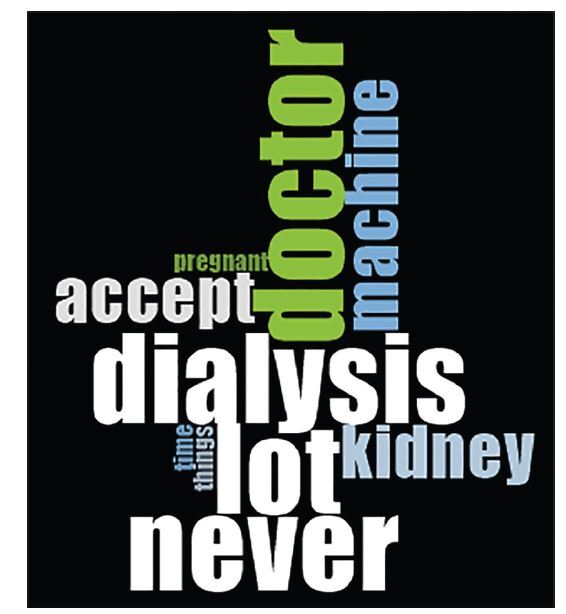
Word cloud based on NVivo analysis of word frequency. NVivo 11 (QSR International MA, USA).

Through the analysis carried out by Nvivo11, we verified that all the interviews analyzed appear in the three categories, so we organized and validated the categories that had been created (Figure 2).

### 1) ASSOCIATION OF PREGNANCY WITH CKD

In the accounts of their life history, the interviewees suggested some important connections between the reproductive event and CKD. Most of them reported having had hypertension during pregnancy and kidney involvement as a consequence.

*I6: I had preeclampsia when I was pregnant… I went off course. With super bad blood pressure… Then, when I realized, I had already lost my kidneys. It had already affected my kidney.*

Some interviewees reported that they had been warned of kidney problems by the obstetricians during pregnancy, but, at that moment, they did not understand the severity of the situation and the risk of unfavorable prognosis.

*I3: I already had it, but I didn't know. Because, since I was a child, my mother has said I was not very healthy. Then, when I got pregnant at 18, they found out that my kidneys had stopped.*

*I5: In the second [pregnancy], I had… I had eclampsia, right? Then, the doctor said: “Look, she has a small kidney problem”. But I didn't pay any attention; he asked me to take some medicine, but I didn't pay attention… And it got worse, right?*

The results reveal that by detailing women's reproductive life histories, it becomes clear how important the period of pregnancy and childbirth is with regards to CKD and how effective and sensitive the communication of the health team needs to be given women's emotional experiences during pregnancy, childbirth, and the postpartum period.

The interviewees also spoke about their desire for motherhood and their doubts and fear regarding CKD and pregnancy. They often cognitively understood the risks and benefits but demonstrated that there are also socio-emotional desires that must be heard, welcomed, and monitored by the health team. Interviewee 4, for example, had CKD since adolescence and says that she always dreamed of becoming a mother. And despite the risks, she became pregnant and had her baby while on dialysis treatment.

*I4: My husband is always afraid, as usual… afraid of losing me. So in the consultation with the gynecologist, he said more than once “Think carefully, think carefully” … as always, right? So, I gave up. But after some time…. I was pregnant… I was three months pregnant! And for me that was everything. I didn't want to hear about anything else, only… I was going to take more care of myself than ever, right? Ah… it was difficult, but I’m not sorry, I would do it all again… Even the doctor cried with me!*

Interviewee 2, who lost a kidney and had a stillbirth in her second pregnancy, reported that she really wanted to know and think about the possibility of another pregnancy. Often, healthcare workers’ responses are objective and do not address the subjective anguish of women, including grief over possible infertility.

*I2: I also wanted to know if it was possible… about pregnancy and hemodialysis! For who does hemodialysis… I would really like to [get pregnant again] But I’m afraid they will say that I can't, that it's dangerous, right? Then it's scary… ah, because I have a gynecologist and she says it's not good for me to get pregnant…*

We realize that these statements demonstrate how complex and profound the meaning of pregnancy and motherhood is for some of these women, and how much it is necessary to talk to these women about this subject, not only to inform them about the risks and benefits but to support them emotionally, to take into account their desires and anxieties, and to help them make more careful, conscious, and respectful decisions regarding their sexual and reproductive lives.

### 2) NEBULOSITY IN RELATION TO DIAGNOSIS AND REPRODUCTIVE HISTORY

The results analyzed show that the interviewed women perceive some important connections between some reproductive events in their lives and CKD. However, the analysis also revealed that the causes of kidney problems and even some indicators such as high blood pressure or kidney problems in pregnancy or postpartum seem to be unknown to most of the interviewees.

The term “nebulosity” in this category was chosen for being synonymous with unintelligibility, incomprehensibility. We perceive it as a reason for much anxiety in those women, who were trying on their own to construct a narrative about what had happened to them both in relation to kidney disease and problems in pregnancy. As interviewee 4 said:

*I4: Well, I was twelve, I was at a party… there were firefighters, who liked to throw water on us… Then they wet us. I came home soaked and there it started… Cold, sore throat… My skin was badly infected, then it eventually went to the kidneys and I couldn't control the infection… It stopped in the kidneys once and for all. Both…*

The lack of clear and explanatory communication when the disease is identified – in general by emergency service teams, not experts – together with poor understanding also make it difficult for women to further investigate and care vis-à-vis for their disease:

*I6: Yes… Information was lacking! Because, at the hospital, when I gave birth to my son, they should already… “Hey, we are going to refer you to such and such place to check what this is!” They didn't… They only told me about preeclampsia and nothing about the kidney… I had preeclampsia: “Hey, we are going to refer you to check why…”, isn't it? They didn't provide that.*

*I11: Nobody explained why and how it happened?… No, they never told me… They didn't warn me I was sick… They put me on the machine… They didn't tell me anything!*

We emphasize that our results show that medical teams should be careful when communicating the severity of kidney disease because of the risk of treatment non-adherence. The interviewees demonstrated that they felt anxiety, guilt, and anger for not understanding the severity of their disease, for not starting treatment early, or for not been warned by the physicians as they would have liked.

*I8: Not possible! I didn't have… I didn't have any idea!… I thought it was… like mere high blood pressure and just that… I never imagined I could lose my kidney.*

The diagnoses were also nebulous, according to the interviewees. Some of them felt they were victims of medical errors or delays. Their accounts also reveal the importance of a specialized team for diagnosis.

*I4: Because I started to swell too much, … I had so much pain in my tummy. I went to the doctor, but they said I had worms, anemia… And it didn't get better!… They never talked about doing another, more thorough test… As I arrived there very frail, they transferred me to another town… I arrived at the hospital already unconscious and went straight to intensive care. They did a biopsy and immediately found out. They straight out said it was a kidney thing. It was then that everything began…*

In these reports, it is evident how communication is impaired from the women's perspective, especially about kidney functions in the body and the dialytic treatment.

*I1: I had no information at all, I didn't even know what a dialysis meant. I arrived at the hospital with the result and I started to do dialysis through the catheter; they had already done my fistula and after forty days, I started through the fistula, but I had no idea about anything. Then, as time goes by, we start to realize, don't we? We talk, we realize…*

*I11: Yes, to orientate a lot, because I didn't know I had that!… I learned after I came to the machine because I didn't know! I didn't know! I didn't know what a kidney was!*

The women interviewed belong to lower socioeconomic classes and therefore live in more vulnerable conditions. Moreover, many of them lived in poorer regions of the country or in places where healthcare services are scarcer. Their accounts demonstrate that socioeconomic aspects affect their access to health and might have an impact on the diagnosis process. This perspective is also present in strategies to prevent kidney disease.

*I3: My mother said I had… That I swelled, but, as I lived on a small farm… They… she cured me with medicine, a healer, this kind of things… Then, after 18, I found out that I had a [damaged] kidney… Then, during pregnancy, I had problems, but as I told you I would go to the healer only. On the day the baby was born I got worse. Then, I went to the doctor. When I arrived, he said: “Hey, I think your child died!”. Then, my blood pressure was very high… He said to my family that I wouldn't “escape”!*

A recurring theme among the interviewees who had had CKD since childhood/adolescence was that they “did not want” to treat the disease in adolescence. According to them, they were in denial about the problem when they were young. These statements were followed, in almost all cases, by an account of a deep sense of regret. Their reports, permeated with self-blame and guilt, reveal the social and affective vulnerability they have experienced since adolescence.

*I4: I had to take… Yes, I was monitored, took medicine… Then, the doctor said: “Hey, if you don't treat… You go to the machine! You go to dialysis!”. Then, we enter adolescence… And we don't want to treat anymore, you know? Then, I stopped… It was when it harmed me… And the doctor would warn me and say: “Hey, it'll happen if you don't treat it…”. But we… It doesn't enter our mind, it doesn't. That phase when we are, we are rebels. I would say: “Oh, he's lying…”.*

**Figure 3.**
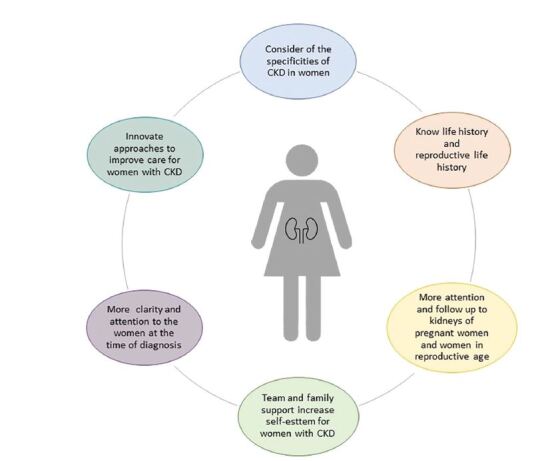
Main themes found in health promotion for women undergoing hemodialysis.

We perceive that some accounts are confused about time and facts. For this reason, our results point to the fact that it is important to provide respectful care to women (Figure 3), with the aim of tracking their overall health status and providing preventive care, as expressed by I2:

*I2: It's complicated! Because it's a silent disease indeed!*

### 3) BEING A WOMAN UNDERGOING HEMODIALYSIS

#### SUBJECTIVE MARKS

The interviews and analysis revealed the fragility and suffering experienced by these women in relation to CKD. There are subjective marks that the interviewees find difficulty to deal with. When they tell about a suffering, they immediately must show and reaffirm to themselves what strength they possess. It is as if they have the simultaneous desire to talk about the pain and not talk about it. They experience the ambivalence and the need to make the most of the present, one day at a time. We perceive the need to defend themselves emotionally and to try to live life “as normal as possible”, even though this “normal” has already been affected and altered by the disease and its treatment, hemodialysis.

*I1: What can I do? There's nothing to be done. You know your life is there in the machine and so you just have to... do what must be done. As I always say. Sometimes I’m not feeling well and someone passes by and says “Are you alright?”. “I’m fine, thank God” …I’m always fine, thank God! Even if I’m dying, I’m alright. [Laughs]*

When talking about their disease, the women report losses and feelings of grief due to CKD. There are the pregnancy losses (miscarriages) and the lost freedom to come and go, to eat, to see people who lived far away and whom they were unable to visit because of hemodialysis.

*I3: I think like this, one thing we can't do is to travel, you know? Not even to my mother. And I have …since I started dialysis, I haven't been back to my mother´s house, my father never came here and died without me being able to see him, after 20 years. I never got to see him.*

*I3: It was really hard when my kidney stopped functioning, you see? Because you don't want that... to lose a child... Then you had that... then I spent 20 years dreaming of a child's crying in my head. Even now... I feel like crying. (Laughs)*

Besides the loss of loved ones and of the life they once lived, they are always feeling that they have to live with uncertainty and fear of their own death.

*I1: We never know what tomorrow will bring.*

*I4: He is my victory, for me [the son] ... I can already... If he wanted to leave, there would be no... Because I have a lot to leave behind... for my mother, right? For my husband, a little piece of me... A continuation. I can leave quite calmly. [Crying]*

#### THE HEMODIALYSIS

The routine of hemodialysis involves considerable organization in women's lives. Many of them are mothers and the mainstay of the family, both in terms of affection and financially. They feel that the illness directly affected their place in the family and do their best to at least maintain their place in affection and childcare, especially the routine of providing meals and cleaning the house. It is as if they feel that hemodialysis could threaten their social role and identity. “In the beginning, it was so difficult to accept...” – this was a fairly recurrent theme among the interviewees, who report how difficult it was to accept hemodialysis, but with time they perceived that “there is nothing I can do about it, right?”. This is resignation, not acceptance.

*I1: My life is in the machine.*

*I9: I miss everything... I even liked to work. I worked in the shopping center, making pizzas. I really liked my job. Then I was unable to work because I felt so bad, right…*

The interviewers formulated questions about gender differences and about social roles, that it is generally the woman who does the housework. It is important to address these specific topics experienced by women to facilitate adherence to hemodialysis treatment and promote a better quality of life and emotional health.

*I1: Yes. Men find it more difficult to accept. I think women accept [it] more. So much that there have been separations, right? Because the man doesn't accept a woman with a problem. Not my husband, my husband, whatever he can do for me he does.*

*I1: Yes, when I began, I did all my work… I worked as usual, and at the time to go to dialysis I would go to dialysis. But today, I can't do that, because things change, you know? Cleaning, things like that, I don't do, but I still wash my clothes, cook, iron. But cleaning and things like that I can't, because of my arm.*

#### OF THE DISEASE, APPEARANCE, AND SELF-ESTEEM

The interviewees talked about issues regarding their femininity and self-concept before the disease, which may leave concrete marks in their body and appearance.

*I3: Well, it was suffering... You know, I suffer until today, because... I don't know if it is the dialysis... with the face of a very old woman, then, my family, all of them beautiful, different, and I ravaged, you know? We get sad with that, don't we? [Laugh]*

*I10: I got very ugly. This part here was full of water, this part here was full of water... Horrible. The more I cried, the more monstrous I was!*

The women interviewed report that, to face so much suffering, they need strength and considerable fight. Some of them even say they that feel like writing the story of their struggle. They feel strong and victorious, despite their suffering.

*I3: A real lesson in life. Because it's not easy, right? To know that you have to be on the machine three times a week... It's not easy. But it's like I said, it's... We know we need it, right? So, we have to fight!*

*I3: Like me, if I was to give up everything... Because sometimes I wanted to give up, then, there was no more I! But I... I fight, and thank God, it's a victory!*

#### SUPPORT

Many women reported how important family support is and how it helped their health. The software Nvivo11 identified the words family and children as two words with high frequency in the interviews.

*I1: But, thank God, my daughter also... she had five children! Then, they are... They are my reason to live. The eldest is nineteen and the youngest is four.*

The interviewees report that they consider the health care team and the other patients who attend the clinic where they undergo hemodialysis as family. They emphasize the importance of the bonds established in the clinic. Family interactions, romantic relationships, and strong friendships arise from this exchange with other people under hemodialysis.

*I4: The personnel here have never seen me crying, it's... sad... I try to rely on my friends here, you know... Everyone has helped me a lot.*

*I7: I was in agony at home; I wouldn't accept it in any way! I didn't want to accept! Then, it was when the same doctor who today will come with me, who I wouldn't like to be far from me... she helped me a lot. It was she who... She came to talk to me; she came to explain a lot of things to me...*

## Discussion

Our study has shown that we should consider the specificities of women undergoing hemodialysis, the issues of their life history allow us to enhance the integral health of these women, including aspects of mental health. Category 1 shows that women make a connection between their reproductive life, especially pregnancy and puerperium, and CKD. The interviewees reported that when they received the news about kidney damage during pregnancy, childbirth, or puerperium they often did not understand the severity or found the information confusing. Our analysis reveals that the health team needs to have a better understanding of how to communicate and guide women who have kidney problems during pregnancy, childbirth, and the postpartum period. This is because these periods involve many physical, biological, psychological, and social changes to women's lives. The preparation and clinical management by the health team at this time are essential. You need to make sure that the woman understood what she was told about her kidney care and that referrals were followed. Our results are consistent with the available literature, confirming that pregnancy plays an important role in the development of CKD and that CKD is under-recognized in women and self-reported awareness about this problem remains low. Similar to our study, Ashuntantang et al.^6^ point out that the important roles women play in society and family life and the health of their kidneys deserve special attention.

Our study highlights the importance of healthcare teams knowing the specificities of women's health to achieve better adherence to their kidneys and mental health care. Also, gynecologists should refer the diagnosed or suspected cases during pregnancy and after birth for a specific follow-up or even during gynecological follow-up for other reasons.

Category 2 reveals how part of the experiences of these women were not understood by themselves. The analysis revealed that this happens mainly for two reasons: 1) lack of communication, effective management, and monitoring by the health team and 2) the emotional experiences of these women in relation to the pregnancy/birth/postpartum process, in addition to the problems related to kidney health, can be very drastic and require the follow-up of a qualified professional who can deal with the emotional health demands and address the anxieties and fears of reconciling motherhood and a health problem.

The third category brought some elements of the subjectivity of the interviewees about the treatment of CKD and their interpersonal relationships. The content analysis made it possible to extract elements from the experiences of these women that can sensitize health professionals to look at the reproductive issues of these women and prepare the health team in approaching and communicating effectively with these women.

The interviewees revealed that it is possible to remain empowered and with high self-esteem regarding their treatment and family relationships when they are supported by the healthcare team and by their families. CKD, as other chronic diseases, may bring changes to the identity of the affected person. This study has also shown that the women interviewed identified that CKD had a direct impact on their professional lives and their physical appearance and self-esteem. Piccoli et al.^1^ state that it is critical for women to maintain their health not only for themselves, but also for their children, family, and society. Women with kidney problems may miss multiple days of work and be less able to care for their children. Perhaps most importantly, their kidney disease and hemodialysis treatment may impact the health of future generations^6^.

It is important to emphasize that our study reveals that CKD isn't only silent, but also very often burdens the life of to the affected woman with a sense of confusion about the moment of diagnosis and the possible causes. Therefore, some periods of life, such as pregnancy and the postpartum period, are relevant for adequate diagnosis and referral of early cases^20^. Piccoli et al^21^ state that “Pregnancy is a unique state for women, offering an opportunity for diagnosis of kidney disease, but also a state where acute and chronic kidney diseases may manifest themselves, and which may impact future generations with respect to kidney health”. The classical example the authors point out is that, during pregnancy, preeclampsia may cause acute kidney damage in women of reproductive age and lead to subsequent CKD; however, the causes for this risk are yet to be fully known^22^. In our study, we also found a lack of investigations regarding predictors of women's reproductive health and CKD.

A survey of nephrologists carried out in North America^23^ found that participants lacked confidence in counseling and caring for women's health. Over 65% of interviewees lacked confidence in counseling their patients about women's health problems, including menstrual disorders, preconception counseling, pregnancy management, and menopause. That same survey indicated that innovative approaches are necessary to improve care for women with kidney disease. Another study, which evaluated the frequency and comprehensiveness of documentation of reproductive health counseling provided by nephrologists to women of childbearing age with CKD attending kidney clinic, reveals a large unmet need in nephrology practice and suggests a closer collaboration with primary care and maternofetal medicine professional and implementation of clinical practice guidelines^24^.

This qualitative study has some limitations. The first is that, despite being an intentional and homogeneous sample, there is some diversity among the women. This has an impact on the differences in the life experiences related to reproductive life. In this aspect, we sought to give voice to the differences by showing them the results. The second limitation is a lack of published studies within the scope of women's health and nephrology, especially concerning quantitative and qualitative indicators of the association of pregnancy with CKD.

This study brings results and discussions from an exploratory qualitative research that sought to identify relevant themes for promoting the health of women with CKD that healthcare teams, especially gynecologists, obstetricians, and nephrologists, should consider when providing support and care to people with this condition. In our view, these are also themes that should be better evaluated and studied in the future to investigate in depth the relationship between women's reproductive life and CKD.

### Implications for Clinical Practice

– Interviews with women undergoing hemodialysis highlight the specifics of these women's feelings and experiences regarding their reproductive life and indicate the importance of innovating approaches to improve care for these women.
– The women undergoing hemodialysis report a history of loss and feelings of mourning. More attention should be given to the women at the time of diagnosis.
– The assistance and attention provided by the health teams to women with CKD should be improved and women in reproductive age require more attention and follow-up.

Our results highlight the importance of the healthcare team addressing the subject of reproductive health with women undergoing hemodialysis because they report their anguish about the possibility of not being able to get pregnant or wanting to know if they will be able to have more children. This team can curb these feeling and help them bear the anxiety and face their condition. The question of reproductive life seems to us to be central in the lives of these women and for this reason needs to be approached and discussed.

## Conclusions

Our research not only provides information about health of women undergoing hemodialysis, but also shows the importance of the healthcare team and healthcare system considering women's life history and prior illnesses. For this, it is important to have multidisciplinary teams and a holistic view of women's health. These aspects make it possible to promote the integral health of women undergoing hemodialysis, including also mental health aspects.
